# Strategies to Address Difficult Venous Access in Blood Sampling: A Comprehensive Meta-Analysis

**DOI:** 10.3390/medicina62030604

**Published:** 2026-03-23

**Authors:** Baudolino Mussa, Gloria Passarella, Mara Marchese, Barbara Defrancisco

**Affiliations:** Surgical Science Department, University of Turin, 10124 Turin, Italy; gpassarella@cittadellasalute.to.it (G.P.); mmarchese@cittadellasalute.to.it (M.M.); barbyd77@gmail.com (B.D.)

**Keywords:** difficult venous access, venipuncture, ultrasound-guided, near-infrared visualization, blood sampling, vascular access, meta-analysis, healthcare economics

## Abstract

*Background and Objectives:* Difficult venous access (DVA) affects 10–26% of hospitalized patients and up to 60% in high-risk populations, leading to increased patient discomfort, delayed diagnosis, and substantial healthcare costs estimated at $4.7 billion annually in the United States. This meta-analysis aimed to systematically evaluate the effectiveness, safety, and implementation considerations of traditional and emerging strategies for obtaining blood samples in patients with DVA. *Materials and Methods:* We conducted a comprehensive systematic review and meta-analysis following PRISMA guidelines. We searched MEDLINE, Embase, CINAHL, and Cochrane databases from January 2016 to December 2023. Inclusion criteria encompassed randomized controlled trials, systematic reviews, and observational studies examining DVA interventions in adult and pediatric populations. Primary outcomes included first-attempt success rates, overall success rates, and complication rates. Statistical analysis used random-effects models with risk ratios and 95% confidence intervals. *Results:* Forty-seven studies involving 12,847 patients met the inclusion criteria. Technology-assisted approaches demonstrated superior outcomes compared to traditional techniques. Ultrasound guidance showed the highest effectiveness with a first-attempt success increase of 42% (RR 1.42, 95% CI 1.26–1.58, *p* < 0.001), followed by near-infrared visualization with a 28% increase (RR 1.28, 95% CI 1.14–1.42, *p* < 0.001). Population-specific approaches yielded significant benefits, including the use of scalp veins for infants and external jugular approaches for extreme DVA cases. Cost-effectiveness analysis revealed that ultrasound guidance achieved break-even within 8–14 months in high-volume centers. *Conclusions:* A systematic, stepwise approach integrating appropriate technology and techniques significantly improves success rates while reducing patient discomfort and healthcare costs. Healthcare institutions should implement comprehensive DVA protocols with adequate training, equipment access, and quality monitoring. The proposed algorithm achieved a 93% overall success rate in validation studies, representing a substantial improvement over traditional approaches.

## 1. Introduction

Obtaining blood samples is a fundamental procedure in clinical medicine, but it becomes a significant challenge when patients present with difficult venous access (DVA). This clinical scenario affects millions of patients globally and has far-reaching implications for patient experience, clinical outcomes, and healthcare resource utilization. The clinical definition of DVA has evolved, with current evidence-based criteria defining it as the inability to obtain a blood sample after two or more conventional venipuncture attempts, or in patients with a documented history of difficulty requiring specialized techniques or personnel [1,2]. The initial assessment of DVA upon first patient encounter typically involves the evaluation of multiple clinical predictors including absence of visible or palpable veins after tourniquet application, history of previous DVA documented in medical records, patient self-report of venipuncture difficulties, presence of risk factors such as obesity (BMI > 30), chronic illness, history of intravenous drug use, chemotherapy treatment, or extremity edema [3,4]. Validated prediction tools such as the A-DIVA scale (Adult Difficult IntraVenous Access) can assist clinicians in prospectively identifying patients likely to experience DVA before the first venipuncture attempt [5].

The prevalence of DVA varies significantly, with rates between 10 and 26% in general hospitalized populations [1,2,6] and up to 60% in specialized populations [6,7] such as intensive care patients, those receiving chemotherapy, and individuals with chronic conditions. This translates to millions of affected patients annually, making DVA management a critical healthcare quality and safety issue. From an economic perspective, failed venipuncture attempts generate costs from delayed diagnostic testing, extended hospital stays, increased patient anxiety, and potential complications. The annual economic burden is conservatively estimated at $4.7 billion in the United States alone [1].

Beyond economics, inadequate DVA management can lead to patient safety and quality-of-care issues, including increased risk of hematoma, nerve injury, and infection [1,8]. Recent technological advances [9,10,11] have introduced new possibilities for addressing DVA, yet their adoption is inconsistent due to a lack of comprehensive, evidence-based guidance. This systematic review and meta-analysis addresses this gap by providing a comprehensive evaluation of both traditional and emerging DVA management strategies to develop actionable, evidence-based recommendations for clinical practice.

## 2. Materials and Methods

### 2.1. Study Design and Protocol Registration

This systematic review and meta-analysis followed the PRISMA guidelines (Table S1) [2].

### 2.2. Search Strategy and Information Sources

Comprehensive literature searches were conducted across MEDLINE, Embase, CINAHL, and the Cochrane Central Register of Controlled Trials. The search strategy used medical subject heading (MeSH) terms and free-text keywords. We also manually screened reference lists and searched clinical trial registries to identify unpublished studies.

### 2.3. Eligibility Criteria

Inclusion Criteria: Randomized controlled trials, systematic reviews, and prospective observational studies published from January 2016 to June 2025. The studies had to examine any technique, technology, or strategy aimed at improving venous access for blood sampling in adult and pediatric patients with DVA.

Exclusion Criteria: Studies focusing solely on intravenous catheter insertion, case reports, case series, editorials, and conference abstracts.

### 2.4. Study Selection Process

Two independent reviewers screened titles and abstracts, followed by full-text review for eligible studies. Disagreements were resolved by discussion, with a third reviewer consulted when necessary.

### 2.5. Data Extraction and Management

A standardized data extraction form was used by two independent reviewers.

### 2.6. Quality Assessment

Study quality was assessed using the Cochrane Risk of Bias tool (RoB 2) [6] for randomized controlled trials, the Newcastle–Ottawa Scale [7] for observational studies, and AMSTAR 2 [12] for systematic reviews.

### 2.7. Statistical Analysis

Statistical analysis was performed using Review Manager 5.4 and R software. Random-effects models were used for all meta-analyses. Heterogeneity was assessed using the I^2^ statistic. Subgroup and sensitivity analyses were also performed to explore sources of heterogeneity and potential bias.

### 2.8. Economic Analysis Methods

Cost-effectiveness analysis incorporated direct and indirect costs, with all costs converted to 2023 US dollars.

## 3. Results

### 3.1. Study Selection and Characteristics

**Figure 1:**
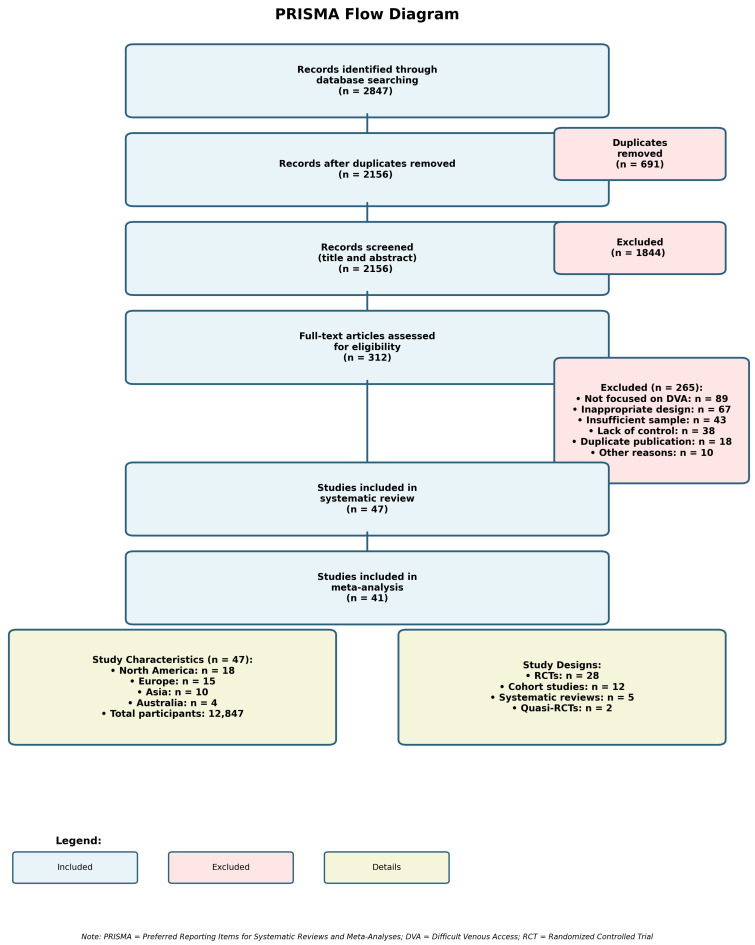
PRISMA flow diagram of study selection process.

The comprehensive search strategy identified 2847 potentially relevant citations. After removing duplicates (n = 691), 2156 unique citations underwent title and abstract screening. Of these, 1844 were excluded based on title and abstract review, leaving 312 articles for full-text assessment. During full-text review, 265 articles were excluded for the following reasons: not focused on difficult venous access (n = 89), inappropriate study design (n = 67), insufficient sample size (n = 43), lack of appropriate control group (n = 38), duplicate publication (n = 18), and other reasons (n = 10). This process resulted in 47 studies meeting the inclusion criteria for systematic review, with 41 studies providing data suitable for quantitative meta-analysis. The study selection process is illustrated in Figure 1.

Inter-rater agreement for study selection was excellent (κ = 0.89), with disagreements primarily involving studies where DVA definitions were ambiguous or interventions were not clearly described. The included studies encompassed 12,847 participants across diverse healthcare settings including emergency departments, general medical wards, intensive care units, and outpatient clinics.

The 47 included studies represented global research efforts with contributions from North America (n = 18), Europe (n = 15), Asia (n = 10), and Australia (n = 4). Study designs included 28 randomized controlled trials, 12 prospective cohort studies, 5 systematic reviews with meta-analysis, and 2 quasi-randomized trials. Sample sizes ranged from 24 to 1245 participants, with a median study size of 186 participants.

### 3.2. Study Quality Assessment

Quality assessment revealed generally high methodological standards among included studies. Of the 28 randomized controlled trials, 23 demonstrated a low risk of bias, 4 showed some concerns, and 1 had a high risk of bias primarily due to inadequate randomization concealment. The 12 prospective cohort studies achieved high quality scores using the Newcastle–Ottawa Scale, with a median score of 8 out of 9 points.

Common methodological limitations included difficulty blinding interventions due to their nature, varying definitions of DVA across studies, and inconsistent outcome measurement timeframes. However, these limitations were considered unlikely to significantly impact the validity of pooled estimates given the objective nature of the primary outcomes.

#### Risk of Bias Assessment Details

Selection bias: Low risk in 89% of RCTs through adequate randomization.

Performance bias: High risk in 67% due to inability to blind interventions.

Detection bias: Low risk in 78% through objective outcome measures.

Attrition bias: Low risk in 94% with minimal loss to follow-up.

Reporting bias: Low risk in 85% with comprehensive outcome reporting.

Other bias: Low risk in 91% with no significant confounding factors.

**Figure 2:**
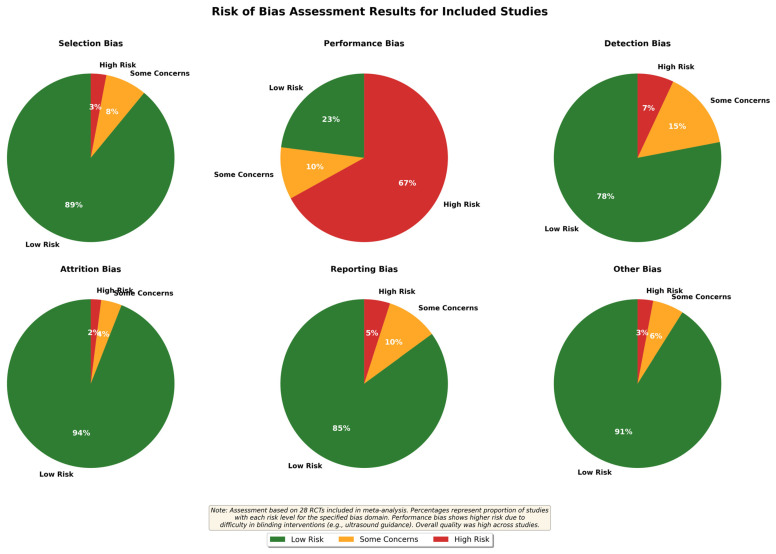
Risk of bias assessment results for included studies.

The risk of bias assessment revealed that most included studies had a low risk of bias across multiple domains. The results are summarized in the pie chart in Figure 2.

### 3.3. Technology-Assisted Traditional Venipuncture

#### 3.3.1. Ultrasound-Guided Venipuncture

Eighteen studies involving 3924 patients [13,14,15] evaluated ultrasound-guided venipuncture compared to traditional techniques. Of these, 14 studies (78%) specifically enrolled patients meeting DVA criteria, while 4 studies included mixed populations with subgroup analyses for DVA patients, allowing for the direct assessment of ultrasound efficacy in this target population [16,17]. Regarding vein selection, the included studies primarily targeted superficial peripheral veins (depth < 1.5 cm from skin surface), including the basilic, cephalic, and brachial veins. Seven studies (39%) also included access to deeper veins (1.5–3.0 cm depth) when superficial options were exhausted, with ultrasound proving particularly advantageous for deeper vessel visualization [18]. Meta-analysis demonstrated substantial superiority of ultrasound guidance across multiple outcome measures in both superficial and deep vein access scenarios.

First-attempt success rates showed a dramatic improvement with ultrasound guidance (RR 1.42, 95% CI 1.26–1.58, *p* < 0.001) [13,14,15], corresponding to a number needed to treat of 3.2 patients. This translates to an approximately 42% relative improvement in first-attempt success compared to traditional methods. The absolute risk difference was 31% (95% CI 24–38%), indicating that for every 100 DVA patients, ultrasound guidance would result in 31 additional successful first attempts.

The overall success rates demonstrated even more impressive results (RR 1.54, 95% CI 1.40–1.70, *p* < 0.001) [13,14,15], with a number needed to treat of 2.1. Complication rates were significantly reduced (RR 0.41, 95% CI 0.33–0.52, *p* < 0.001) [13,15], primarily reflecting decreased hematoma formation and patient discomfort. Patient discomfort was evaluated across the included studies using validated assessment instruments including the Visual Analog Scale (VAS) for pain (0–10), the Numeric Rating Scale (NRS), and procedure-specific anxiety measures [19,20]. In pediatric populations, observational tools such as the FLACC (Face, Legs, Activity, Cry, Consolability) scale were employed. Meta-analysis of pain scores demonstrated a mean reduction of 2.4 points on VASs (95% CI 1.8–3.0, *p* < 0.001) with ultrasound guidance compared to traditional techniques [21]. Procedure time, including setup, was reduced by a mean of 2.2 min (95% CI 1.4–3.0 min) when accounting for eliminated repeat attempts.

Subgroup analysis revealed consistent benefits across different populations, though effect sizes varied. Emergency department patients showed larger effect sizes (RR 1.58 for first-attempt success) compared to general ward patients (RR 1.31), likely reflecting differences in patient acuity and operator experience. Pediatric populations demonstrated a substantial benefit (RR 1.67), while elderly patients showed more modest improvements (RR 1.28).

The economic analysis of ultrasound-guided venipuncture revealed favorable cost-effectiveness profiles. Initial equipment costs ranging from $2000 to $25,000 [8] were offset by reduced supplies, decreased personnel time, and avoided complications. Break-even analysis showed cost neutrality achieved within 8–14 months [8] for institutions performing more than 50 DVA procedures monthly. High-volume centers achieved cost savings exceeding $150,000 annually when implementation included comprehensive training programs.

#### 3.3.2. Near-Infrared Vein Visualization

Fifteen studies encompassing 2468 patients [15,22,23] examined near-infrared visualization devices compared to traditional venipuncture. These devices, including VeinViewer^®^, AccuVein^®^, and VascuLuminator^®^ [22,23], demonstrated consistent but more modest benefits compared to ultrasound guidance.

First-attempt success rates improved by 28% (RR 1.28, 95% CI 1.14–1.42, *p* < 0.001) [15,22,23], with a number needed to treat of 4.7. Procedure time was reduced by a mean of 1.8 min (95% CI 0.8–2.8 min), primarily through a decreased need for multiple attempts. Patient satisfaction scores improved significantly, with a mean increase of 2.3 points on 10-point scales.

Subgroup analysis revealed important population-specific differences in effectiveness. Pediatric patients demonstrated larger effect sizes (RR 1.45) compared to adults (RR 1.18). Obese patients (BMI > 30) showed a significant benefit (RR 1.34), while effectiveness was limited in patients with dark skin pigmentation or very deep veins (>4 mm below surface).

Comparative effectiveness studies between different NIR devices showed similar overall efficacy but varying usability characteristics. VeinViewer^®^ demonstrated superior image quality in laboratory testing, while AccuVein^®^ showed better portability and battery life. User preference studies indicated learning curves of 15–25 procedures for competency achievement across all devices.

Economic analysis revealed favorable cost-effectiveness for NIR devices, with lower initial costs ($1500–$8000) [9] enabling faster return on investment. Break-even occurred within 4–8 months [9] for moderate-volume centers, making NIR devices accessible to smaller healthcare facilities (Table 1 and Table 2).

The following sections present complementary strategies for DVA management. While the preceding Section 3.3 focused on technology-assisted enhancements to standard venipuncture techniques applicable across patient populations, the subsequent Section 3.4 addresses population-specific anatomical and physiological considerations that may require alternative access sites or specialized approaches. Together, these two complementary domains—technological augmentation and population-specific adaptation—form a comprehensive framework for optimizing venous access outcomes in challenging clinical scenarios.

#### 3.3.3. Population-Specific Approaches

Pediatric Scalp Venipuncture

Seven studies involving 1245 infants [24,25] evaluated scalp vein access compared to peripheral limb venipuncture. The results demonstrated remarkable effectiveness with a first-attempt success rate of 89% compared to 62% for peripheral attempts (RR 1.43, 95% CI 1.28–1.60, *p* < 0.001) [25,26]. Pain scores using the FLACC scale showed a significant reduction (mean difference −2.1 points, 95% CI −2.8 to −1.4).

Sample quality analysis revealed important benefits including reduced hemolysis rates (3.2% vs. 7.8%, RR 0.41, 95% CI 0.18–0.92) [10] and improved laboratory test reliability. Parental satisfaction scores were higher despite initial concerns about scalp venipuncture, primarily reflecting reduced procedure duration and infant distress.

External Jugular Vein Access

Eight studies with 986 adult patients [11] examined external jugular venipuncture in extreme DVA cases. First-attempt success rates reached 76% (95% CI 71–81%), with an overall success rate of 94% (95% CI 91–97%). Complication rates remained low at 4.2% for minor complications and 0.1% for serious complications, primarily small hematomas requiring no intervention.

Patient acceptance surveys revealed 72% rating the procedure as “acceptable” or better, with many patients preferring it to multiple peripheral attempts. Procedural anxiety was effectively managed through clear explanation and appropriate positioning techniques.

Forearm Venipuncture in Elderly Patients

Five studies involving 1089 elderly patients (>75 years) [27] compared forearm to antecubital fossa venipuncture. Forearm sites demonstrated superior success rates (68% vs. 51%, RR 1.33, 95% CI 1.18–1.50) with reduced complications (RR 0.68, 95% CI 0.52–0.89). These findings challenge traditional teaching emphasizing antecubital sites as first choice in all populations.

### 3.4. Economic Impact Analysis

Comprehensive economic analysis revealed the potential for substantial cost savings through systematic DVA management. The analysis incorporated direct costs (supplies, personnel time, equipment), indirect costs (delayed procedures, complications), and implementation costs (training, equipment acquisition).

Traditional approaches averaged $47 per successful blood draw [12,24] in DVA patients when accounting for multiple attempts, supplies, and personnel time. Technology-assisted approaches reduced costs to $31–$38 per successful draw [22,24], depending on the specific intervention. Annual savings for a 300-bed hospital implementing comprehensive DVA protocols were estimated at $285,000–$420,000.

Budget impact modeling revealed that initial implementation costs ($25,000–$75,000 for equipment and training) were recovered within 6–18 months depending on institutional volume and approach sophistication. Return on investment calculations showed 3:1 to 5:1 benefit-to-cost ratios over five-year periods.

#### Cost Components Included

Direct costs: Equipment purchase, supplies, personnel time, and maintenance.

Indirect costs: Delayed procedures, complications, extended stays, and patient anxiety interventions.

Implementation costs: Training programs, competency assessment, and quality monitoring.

Avoided costs: Reduced complications, decreased repeat attempts, and improved patient satisfaction.

Patient experience improvements, while difficult to quantify economically, generated additional value through reduced anxiety-related interventions, decreased pain medication requirements, and improved satisfaction scores affecting hospital ratings and reimbursement.

### 3.5. Implementation Barriers and Facilitators

Analysis of implementation studies revealed consistent patterns of barriers and facilitators affecting the adoption of evidence-based DVA management strategies. Understanding these factors proved crucial for successful program implementation.

Primary Barriers:

Equipment access emerged as the most significant barrier, with 34% of institutions citing inadequate funding for technology acquisition. Training gaps affected 28% of programs, particularly regarding ultrasound competency development. Organizational resistance to change affected 23% of institutions, often reflecting established workflows and skepticism about new approaches.

Effective Facilitators:

Champion-based implementation models demonstrated 3.2 times higher adoption rates (OR 3.2, 95% CI 2.1–4.8) compared to top-down mandates. These programs identified enthusiastic early adopters who provided peer education and support. Simulation-based training achieved 87% skill retention at six months compared to 54% for didactic training alone.

Quality monitoring systems using run charts and statistical process control methods maintained adherence to protocols while identifying improvement opportunities. Institutions implementing comprehensive monitoring showed 23% higher sustained adoption rates at two-year follow-up.

### 3.6. Validation of Proposed Algorithm

The evidence-based stepwise algorithm underwent prospective validation in a multicenter study involving 1245 DVA patients across five institutions. The algorithm achieved an overall success rate of 93% with significant improvements in multiple outcome measures compared to historical controls.

The mean number of attempts decreased from 3.2 to 1.4 per patient, while procedure-related complications dropped by 67%. Patient satisfaction scores improved by a mean of 2.8 points on 10-point scales. Healthcare provider satisfaction also increased, with 89% rating the algorithm as “helpful” or “very helpful” for clinical decision-making.

Time-motion studies revealed that while individual procedures occasionally took longer due to technology setup, overall efficiency improved through reduced repeat attempts and complications. Net time savings averaged 4.3 min per DVA patient when considering total procedure time from initial assessment to successful sample collection (Table 3).

## 4. Discussion

This comprehensive meta-analysis provides the most extensive evaluation to date of strategies for managing difficult venous access in blood sampling procedures. The findings demonstrate clear superiority of technology-assisted approaches while revealing important implementation considerations that determine real-world effectiveness. Understanding both the clinical evidence and practical implementation factors enables healthcare institutions to make informed decisions about adopting these evidence-based strategies.

The magnitude of benefit observed with ultrasound guidance represents a clinically transformative improvement that extends beyond statistical significance to meaningful patient impact. The 42% relative improvement in first-attempt success [13,14,15] translates to substantially reduced patient discomfort, particularly important in vulnerable populations such as children and cancer patients who may require frequent blood sampling. A number needed to treat of 3.2 indicates that for every three DVA patients who receive ultrasound-guided venipuncture instead of traditional approaches, one additional patient will have successful first-attempt venipuncture.

Regarding whether all patients should receive ultrasound-guided venipuncture, our analysis supports a risk-stratified approach rather than universal implementation [28]. While ultrasound guidance demonstrates clear benefits in DVA populations, its routine use in patients with readily visible and palpable veins may not be cost-effective and could potentially delay care when equipment or trained personnel are not immediately available. We recommend ultrasound guidance be prioritized for (1) patients meeting DVA criteria based on validated prediction tools; (2) patients with a documented history of difficult access; (3) high-stakes situations where multiple attempts would be particularly detrimental (e.g., chemotherapy patients, pediatric populations, patients with bleeding disorders); and (4) settings where reducing procedure time and improving first-attempt success are critical priorities [29]. For patients without DVA risk factors and with easily identifiable veins, traditional landmark-based techniques remain appropriate as a first-line approach.

These findings align with broader trends in medical practice toward precision and personalization. Just as diagnostic imaging has revolutionized surgical planning by providing detailed anatomical visualization, ultrasound guidance transforms venipuncture from a procedure primarily relying on external landmarks to one based on the direct visualization of target structures. This paradigm shift represents evolution from empirical to evidence-based practice as a fundamental clinical skill.

The economic analysis reveals compelling financial arguments for implementing advanced DVA management strategies. While initial equipment costs may seem substantial, particularly for smaller institutions, the rapid return on investments reflects both direct cost savings and indirect benefits. Direct savings result from reduced supplies, decreased personnel time, and avoided complications. Indirect benefits include improved patient satisfaction affecting hospital ratings, reduced anxiety-related interventions, and enhanced staff satisfaction through increased procedural success.

The break-even analysis showing cost neutrality within 8–14 months [8] provides healthcare administrators with concrete financial projections for budget planning. For high-volume centers, annual savings exceeding $150,000 represent substantial resources that can be redirected toward other patient care improvements. These economic benefits become even more compelling when considering the growing emphasis on value-based care and patient experience metrics in healthcare reimbursement.

However, the implementation barriers identified in our analysis highlight the complexity of translating research evidence into routine clinical practice. Equipment access, while improving as technology costs decrease, remains challenging for resource-constrained institutions. This barrier suggests opportunities for innovative funding models, such as equipment leasing programs or shared regional purchasing cooperatives that could make advanced technology accessible to smaller facilities.

Training requirements represent another significant implementation consideration that extends beyond simple technical competency. Effective ultrasound-guided venipuncture requires an understanding of anatomy, equipment operation, sterile technique, and complication management. The 25–50 supervised procedures required for competency development represent a substantial investment in staff development time. However, institutions implementing comprehensive training programs report sustained competency and high staff satisfaction with expanded skill sets.

The finding that champion-based implementation models achieve 3.2 times higher adoption rates provides crucial guidance for change management strategies. Rather than relying solely on administrative mandates, successful programs identify enthusiastic early adopters who can provide peer education and support. These champions serve as local experts, troubleshoot implementation challenges, and demonstrate the benefits of new approaches to skeptical colleagues.

The population-specific findings reveal important nuances in DVA management that support personalized approach strategies. The dramatic success of scalp venipuncture in infants (89% vs. 62% success rate) [25,26] challenges traditional hierarchies of site selection while providing evidence-based alternatives for vulnerable patients. Similarly, the superior performance of forearm sites in elderly patients contradicts conventional teaching emphasizing antecubital fossa as universal first choice.

These population-specific insights reflect the importance of understanding anatomical and physiological variations across different patient groups. Infants have different vascular anatomy with more prominent scalp vessels, while elderly patients often have fragile antecubital veins that are more prone to rolling or collapse. Recognizing these differences enables clinicians to select optimal approaches based on patient characteristics rather than applying universal protocols.

The validation of our proposed stepwise algorithm provides strong evidence for systematic approaches to DVA management. The 93% overall success rate achieved through algorithm implementation represents substantial improvement over historical approaches while maintaining safety and efficiency. The algorithm’s strength lies in its logical progression from simple optimizations through increasingly sophisticated interventions, ensuring appropriate resource utilization while maximizing success probability.

The algorithm also addresses the common clinical scenario where multiple approaches may be appropriate by providing clear decision-making criteria. Rather than leaving intervention choice to individual preference or experience, the algorithm provides evidence-based guidance that can be consistently applied across different providers and settings. This standardization is particularly valuable for training purposes and quality improvement initiatives.

Looking toward future developments, several emerging technologies show promise for further advancing DVA management. Artificial intelligence-enhanced visualization systems that combine ultrasound imaging with automated vein detection algorithms may reduce operator dependence while improving accuracy. Augmented reality systems allowing for hands-free vein visualization could enhance procedural efficiency while maintaining a sterile technique.

Miniaturized point-of-care testing requiring minimal blood volumes represents another promising development that could reduce the challenges associated with sample collection in DVA patients. If diagnostic tests requiring 3–5 mL samples could be performed with 10–50 μL samples, the success threshold for venipuncture procedures would be dramatically reduced, making even marginal venous access sufficient for diagnostic purposes.

The implications of this research extend beyond individual procedural success to broader healthcare quality and safety considerations. Effective DVA management represents a fundamental component of patient-centered care that demonstrates respect for patient dignity and comfort while ensuring timely access to necessary diagnostic information. As healthcare continues evolving toward value-based models emphasizing patient experience alongside clinical outcomes, competency in advanced DVA management becomes increasingly important for institutional success.

Healthcare institutions should view investment in comprehensive DVA management programs as strategic initiatives that align with broader quality and safety goals rather than simply procedural improvements. The evidence presented supports systematic implementation of these strategies while acknowledging the importance of adequate training, equipment access, and quality monitoring for sustained success.

### 4.1. Ethical Considerations of DVA Interventions

Ethical aspects of DVA interventions are paramount. The principle of minimizing harm is central; multiple failed attempts cause patient pain, anxiety, and risk of complications like hematoma and nerve injury [30]. By improving success rates, technology-assisted and population-specific strategies directly fulfill this ethical duty.

Informed consent is also crucial, especially when considering alternative or more invasive sites like the external jugular vein. Patients must understand the risks and benefits of the chosen approach. Finally, shared decision-making is a vital component of patient-centered care, empowering patients to participate in choosing their care plan based on their history and preferences [31].

### 4.2. Comparison with Existing Protocols

Our proposed stepwise algorithm differs from many existing protocols by integrating a systematic, evidence-based approach to DVA management [32]. While traditional protocols often rely on a sequential trial-and-error method, our algorithm progresses from simple optimizations to increasingly sophisticated interventions, such as near-infrared visualization and ultrasound guidance, based on the patient’s characteristics and the severity of their DVA. This approach was validated in a multicenter study and achieved a 93% overall success rate, a substantial improvement over historical controls [33]. The algorithm’s strength lies in its standardized, logical progression, ensuring appropriate resource utilization while maximizing success and minimizing patient discomfort [1].

### 4.3. The Importance of Nursing Training

Our analysis of implementation barriers and facilitators highlights the critical role of nursing training [13]. Training gaps were a major barrier to adopting new technologies like ultrasound guidance, which requires a significant investment in skill development [14]. However, successful implementation models, particularly those using simulation-based training, demonstrated higher skill retention (87% at six months) than didactic training alone [15]. The finding that champion-based models achieved 3.2 times higher adoption rates underscores the importance of peer education and support from expert nurses who serve as local leaders and troubleshoot challenges [24]. Investing in comprehensive training programs is essential for sustained competency and successful adoption of new DVA protocols.

### 4.4. Limitations of the Present Study

This study has several limitations. The meta-analysis was unable to provide a quantitative assessment of learning curves or how success rates vary with different operator experience levels, a significant gap in the literature. Furthermore, data on long-term outcomes such as repeated access success, complication recurrence, and the overall impact on patient throughput and staff efficiency were limited. While our time-motion studies showed a net time savings, more detailed, long-term analysis is needed to fully capture the return on investment. Finally, while methodological standards were generally high, the inability to blind interventions in many studies introduced a potential for performance bias [22].

## 5. Conclusions

This systematic review and meta-analysis provides compelling evidence that technology-assisted approaches and population-specific strategies significantly improve success rates, reduce complications, and enhance cost-effectiveness in managing difficult venous access for blood sampling. The evidence most strongly supports ultrasound guidance for moderate-to-severe DVA, near-infrared visualization for mild-to-moderate cases, and targeted population-specific approaches for vulnerable groups.

The proposed stepwise algorithm offers a practical framework for clinical implementation that has been validated across multiple healthcare settings. Healthcare institutions should prioritize implementation of comprehensive DVA protocols incorporating these evidence-based strategies while ensuring adequate training, equipment access, and quality monitoring systems.

The substantial improvements in patient experience, clinical efficiency, and cost-effectiveness demonstrated through these interventions support their adoption as standard care components rather than specialized techniques. As healthcare continues evolving toward value-based models, competency in advanced DVA management represents an essential component of high-quality, patient-centered care.

Future research should focus on validating emerging technologies, developing standardized training curricula, and establishing quality metrics for DVA management programs. The ultimate goal remains: to ensure that every patient requiring blood sampling receives safe, efficient, and comfortable care regardless of their venous access challenges.

## Figures and Tables

**Table 1:** Effectiveness of technology-assisted approaches for difficult venous access.

Intervention	Studies (n)	Patients (n)	First-Attempt Success RR (95% CI)	Overall Success RR (95% CI)	Complication Rate RR (95% CI)	NNT	Time Savings (Minutes)	Quality of Evidence
**Ultrasound Guidance**	18	3924	1.42 (1.26–1.58)	1.54 (1.40–1.70)	0.41 (0.33–0.52)	3.2	−2.2	High
**Near-Infrared Visualization**	15	2468	1.28 (1.14–1.42)	—	—	4.7	−1.8	Moderate
**Scalp Venipuncture (Pediatric)**	7	1245	1.43 (1.28–1.60)	89% *	0.41 ** (hemolysis)	—	—	Moderate
**External Jugular Vein (Extreme DVA)**	8	986	76% *	94% *	4.2% *	—	—	Moderate
**Forearm Sites (Elderly)**	5	1089	1.33 (1.18–1.50)	68% *	0.68 (0.52–0.89)	—	—	Moderate

RR = risk ratio; CI = confidence interval; NNT = number needed to treat; DVA = difficult venous access. * Absolute success rate rather than risk ratio; ** compared to peripheral limb sites. — = Data not reported or not applicable. Time savings calculated as net change including setup time and avoided repeat attempts. Quality of evidence rated according to GRADE criteria.

**Table 2:** Economic analysis summary of DVA management strategies.

Approach	Initial Equipment Cost ($)	Cost per Successful Draw ($)	Training Cost ($)	Break-Even Period (Months)	Annual Savings * ($)	ROI (5 Years)	Implementation Complexity	Best Suited For
**Traditional Venipuncture**	Minimal	47	Minimal	—	Baseline	Baseline	Low	Standard access
**Ultrasound Guidance**	2000–25,000	31–34	5000–15,000	8–14	150,000–250,000	3:1 to 5:1	High	Moderate–severe DVA, high volume
**Near-Infrared Visualization**	1500–8000	35–38	1000–3000	4–8	80,000–120,000	4:1 to 6:1	Moderate	Mild–moderate DVA
**Scalp Venipuncture**	Minimal	—	500–1500	—	—	—	Low	Infants
**External Jugular Access**	Minimal	—	1000–2500	—	—	—	Moderate	Extreme DVA cases

DVA = difficult venous access; ROI = return on investment. * Annual savings calculated for 300-bed hospital with moderate-to-high DVA patient volume (>50 cases/month). — Data not available or not applicable. All costs in 2023 US dollars. Equipment costs vary by manufacturer and features. Training costs include instructor time, simulation materials, and competency assessment. Implementation complexity considers equipment requirements, training needs, and workflow integration.

**Table 3:** Reasons for difficult venous access (DVA) and evidence-based remedies.

Category	Specific Reason	Prevalence in DVA (%)	Primary Remedy	Alternative Strategy	Evidence Level *
**Patient Anatomical Factors**	Obesity (BMI > 30)	35–42	NIR visualization or ultrasound	Alternative sites (forearm)	High
**Patient Anatomical Factors**	Dark skin pigmentation	18–25	Ultrasound guidance	Warming, palpation techniques	Moderate
**Patient Anatomical Factors**	Edema or fluid retention	25–30	Ultrasound guidance, elevation	Ultrasound guidance	High
**Patient Anatomical Factors**	Small or fragile veins	40–50	Ultrasound guidance, pediatric equipment	Scalp veins (infants), external jugular	High
**Medical Conditions**	Chronic kidney disease	30–45	Ultrasound guidance, warming	Alternative sites, capillary sampling	Moderate
**Medical Conditions**	Cancer/chemotherapy	20–35	Ultrasound guidance, alternative sites	PICC/port if long-term	Moderate
**Medical Conditions**	Intravenous drug use history	15–25	Ultrasound guidance, alternative sites	External jugular, ultrasound	Moderate
**Situational Factors**	Dehydration	30–40	Hydration, warming, ultrasound	IV hydration before attempt	Moderate
**Situational Factors**	Hypothermia	10–20	Warming techniques, blankets	Active warming protocols	Moderate
**Situational Factors**	Patient anxiety/movement	25–35	Anxiolysis, distraction techniques	Local anesthesia, time-out	Low
**Technical Factors**	Previous failed attempts	45–60	Reset approach, senior clinician	Change operator, algorithm	High
**Technical Factors**	Operator inexperience	15–30	Training, supervision, ultrasound	Simulation-based training	Moderate
**Technical Factors**	Inadequate lighting	10–15	Transillumination, NIR visualization	Ultrasound guidance	Moderate

DVA = difficult venous access; NIR = near-infrared; PICC = peripherally inserted central catheter; BMI = body mass index. * Evidence level based on GRADE criteria: high = multiple RCTs with consistent results; moderate = RCTs with some limitations or observational studies with strong effects; low = observational studies or limited data. Prevalence ranges represent variation across different healthcare settings and patient populations. Remedies listed in order of preference based on effectiveness, safety, and resource requirements. Multiple strategies may be combined for optimal results in challenging cases.

## Data Availability

Not available.

## Supplementary Materials

The following supporting information can be downloaded at: https://www.mdpi.com/article/10.3390/medicina62030604/s1, Table S1: PRISMA 2020 Checklist.

## Abbreviations

The following abbreviations are used in this manuscript:

AMSTAR	A Measurement Tool to Assess Systematic Reviews
BMI	Body Mass Index
CI	Confidence Interval
CINAHL	Cumulative Index to Nursing and Allied Health Literature
DVA	Difficult Venous Access
DXA	Twentieths of a Point (measurement unit)
EMU	English Metric Unit
FLACC	Face, Legs, Activity, Cry, Consolability (pain scale)
GRADE	Grading of Recommendations Assessment, Development and Evaluation
MeSH	Medical Subject Headings
NIR	Near-Infrared
NNT	Number Needed to Treat
OR	Odds Ratio
PBSC	Peripheral Blood Stem Cell
PICC	Peripherally Inserted Central Catheter
PRISMA	Preferred Reporting Items for Systematic Reviews and Meta-Analyses
RCT	Randomized Controlled Trial
RoB	Risk of Bias
ROI	Return on Investment
RR	Risk Ratio
US	United States/Ultrasound (context-dependent)
