# Supplementary material for: Strategies to Address Difficult Venous Access in Blood Sampling: A Comprehensive Meta-Analysis
Source: Medicina (Kaunas). 2026 Mar 23;62(3):604. doi: 10.3390/medicina62030604 (PMC13027773; doi:10.3390/medicina62030604)
Supplement: Supplementary file 1 [file medicina-62-00604-s001.zip › medicina-4077961-supplementary-Table S1.pdf]

# PRISMA 2020 Checklist

| Section and Topic | Item # | Checklist item | Location where item is reported |
|---|---|---|---|
| **TITLE** | | | |
| Title | 1 | Identify the report as a systematic review. | Title page ("Strategies to Address Difficult Venous Access in Blood Sampling: A Comprehensive Meta-analysis"; "Review" heading; Abstract states "systematic review and meta-analysis"). |
| **ABSTRACT** | | | |
| Abstract | 2 | See the PRISMA 2020 for Abstracts checklist. | Structured Abstract (Background and Objectives, Materials and Methods, Results, Conclusions). Structured Abstract (Background and Objectives, Materials and Methods, Results, Conclusions). |
| **INTRODUCTION** | | | |
| Rationale | 3 | Describe the rationale for the review in the context of existing knowledge. | Introduction, paragraphs 1–3 (burden of |

| | | | |
|---|---|---|---|
| | | | DVA, prevalence, costs, clinical impact, lack of comprehensive guidance). |
| Objectives | 4 | Provide an explicit statement of the objective(s) or question(s) the review addresses. | Abstract (Background and Objectives) and end of Introduction (aims to provide comprehensive evaluation and develop evidence-based recommendations/stepwise algorithm). |
| **METHODS** | | | |
| Eligibility criteria | 5 | Specify the inclusion and exclusion criteria for the review and how studies were grouped for the syntheses. | Materials and Methods – "2.3. Eligibility Criteria" (inclusion and exclusion criteria; study designs; population; time window 2016–2025; focus on techniques/strategies to improve venous access). |
| Information sources | 6 | Specify all databases, registers, websites, organisations, reference lists and other sources searched or consulted to identify studies. Specify the date when each source was last searched or consulted. | Materials and Methods – "2.2. Search Strategy and Information Sources" (MEDLINE, Embase, CINAHL, Cochrane Central; manual reference screening; clinical trial registries; date range January |

| | | | |
|---|---|---|---|
| | | | 2016–June 2025). |
| Search strategy | 7 | Present the full search strategies for all databases, registers and websites, including any filters and limits used. | Materials and Methods – “2.2. Search Strategy and Information Sources” (MeSH terms and free-text keywords; multiple databases and registries). Full line-by-line search strings are not shown; you may want to add these in an appendix. |
| Selection process | 8 | Specify the methods used to decide whether a study met the inclusion criteria of the review, including how many reviewers screened each record and each report retrieved, whether they worked independently, and if applicable, details of automation tools used in the process. | Materials and Methods – “2.4. Study Selection Process” (two independent reviewers, title/abstract and full-text screening, consensus and third-reviewer adjudication); Results – “3.1. Study Selection and Characteristics” and Figure 1 (PRISMA flow). |
| Data collection process | 9 | Specify the methods used to collect data from reports, including how many reviewers collected data from each report, whether they worked independently, any processes for obtaining or confirming data from study investigators, and if applicable, details of automation tools used in the process. | Materials and Methods – “2.5. Data Extraction and Management” (standardized extraction form, two independent reviewers) and mention of contacting |

| | | | |
|---|---|---|---|
| | | | investigators is not explicit (no additional data-request process described). |
| Data items | 10a | List and define all outcomes for which data were sought. Specify whether all results that were compatible with each outcome domain in each study were sought (e.g. for all measures, time points, analyses), and if not, the methods used to decide which results to collect. | Materials and Methods – Abstract "Primary outcomes included first-attempt success rates, overall success rates, and complication rates"; section 2.7 (outcomes for meta-analysis), and Results subsections (e.g., first-attempt success, overall success, complications, pain scores, procedure time, economic outcomes). |
| | 10b | List and define all other variables for which data were sought (e.g. participant and intervention characteristics, funding sources). Describe any assumptions made about any missing or unclear information. | Materials and Methods – sections 2.5 and 2.6 (participant characteristics, settings, intervention type, technology, economic parameters), and Results (study designs, populations, settings, region, cost variables, implementation factors). |

| | | | |
|---|---|---|---|
| | | | Assumptions on missing data are not explicitly detailed. |
| Study risk of bias assessment | 11 | Specify the methods used to assess risk of bias in the included studies, including details of the tool(s) used, how many reviewers assessed each study and whether they worked independently, and if applicable, details of automation tools used in the process. | Materials and Methods – "2.6. Quality Assessment" (RoB 2 for RCTs, Newcastle–Ottawa Scale for observational studies, AMSTAR 2 for systematic reviews; two independent reviewers implied) and Results – "3.2. Study Quality Assessment" and "3.2.1. Risk of Bias Assessment Details" and Figure 2. |
| Effect measures | 12 | Specify for each outcome the effect measure(s) (e.g. risk ratio, mean difference) used in the synthesis or presentation of results. | Abstract (risk ratios with 95% CIs), Materials and Methods – "2.7. Statistical Analysis" (risk ratios, mean differences, 95% CIs, $I^2$), and Results (RRs, absolute risks, NNT, mean differences). |
| Synthesis methods | 13a | Describe the processes used to decide which studies were eligible for each synthesis (e.g. tabulating the study intervention characteristics and comparing against the planned groups for each synthesis (item #5)). | Materials and Methods – "2.3. Eligibility Criteria", "2.4. Study Selection Process", and "2.7. Statistical |

| | | | |
|---|---|---|---|
| | | | Analysis”; Results – “3.3. Technology-Assisted Traditional Venipuncture” and “3.4. Population-Specific Approaches” (grouped by intervention type and population). |
| | 13b | Describe any methods required to prepare the data for presentation or synthesis, such as handling of missing summary statistics, or data conversions. | Materials and Methods – “2.7. Statistical Analysis” (random-effects models, handling of heterogeneity; basic transformations implied) and Results – reporting of converted metrics (e.g., NNT, cost conversions to 2023 US dollars in “2.8. Economic Analysis Methods”). |
| | 13c | Describe any methods used to tabulate or visually display results of individual studies and syntheses. | Materials and Methods – “2.7. Statistical Analysis”; Results – tables and figures (Table 1: effectiveness; Table 2: economic analysis; Table 3: reasons and remedies; Figures 1–2). |

| | | | |
|---|---|---|---|
| | 13d | Describe any methods used to synthesize results and provide a rationale for the choice(s). If meta-analysis was performed, describe the model(s), method(s) to identify the presence and extent of statistical heterogeneity, and software package(s) used. | Materials and Methods – "2.7. Statistical Analysis" (random-effects models for all meta-analyses, I² statistic, use of Review Manager 5.4 and R software). |
| | 13e | Describe any methods used to explore possible causes of heterogeneity among study results (e.g. subgroup analysis, meta-regression). | Materials and Methods – "2.7. Statistical Analysis" (subgroup and sensitivity analyses planned) and Results – multiple subgroup analyses (e.g., ED vs ward, pediatric vs adult, obese vs non-obese, population-specific strategies). |
| | 13f | Describe any sensitivity analyses conducted to assess robustness of the synthesized results. | Materials and Methods – "2.7. Statistical Analysis" (sensitivity analyses planned to explore bias and heterogeneity) and Results – "3.7. Validation of Proposed Algorithm" and economic sensitivity/budget impact analyses in "3.5. Economic Impact Analysis" |

| | | | |
|---|---|---|---|
| | | | (though numerical details of statistical sensitivity analyses are relatively high-level). |
| Reporting bias assessment | 14 | Describe any methods used to assess risk of bias due to missing results in a synthesis (arising from reporting biases). | Materials and Methods – briefly implied under "2.6. Quality Assessment" and "2.7. Statistical Analysis" (reporting bias domain in RoB 2 and NOS; mention of reporting bias in "3.2.1. Risk of Bias Assessment Details"); explicit methods such as funnel plots are not detailed. |
| Certainty assessment | 15 | Describe any methods used to assess certainty (or confidence) in the body of evidence for an outcome. | Results – Table 1 and Table 3 (quality or evidence level rated by GRADE), Methods – implicit in "2.6. Quality Assessment" (stating use of GRADE criteria). |

| Section and Topic | Item # | Checklist item | Location where item is reported |
|---|---|---|---|
| **RESULTS** | | | |

| | | | |
|---|---|---|---|
| Study selection | 16a | Describe the results of the search and selection process, from the number of records identified in the search to the number of studies included in the review, ideally using a flow diagram. | Results – "3.1. Study Selection and Characteristics" and Figure 1 (numbers identified, screened, excluded, included, with reasons). |
| | 16b | Cite studies that might appear to meet the inclusion criteria, but which were excluded, and explain why they were excluded. | Results – "3.1. Study Selection and Characteristics" (aggregated counts and reasons: not focused on DVA, inappropriate design, small sample, lack of control, duplicates, others); individual excluded citations are not listed in a separate table. |
| Study characteristics | 17 | Cite each included study and present its characteristics. | Results – "3.1. Study Selection and Characteristics" (designs, settings, participants, regions, sample sizes) and detailed narrative in subsections 3.3–3.4 and Tables 1 and 3. |
| Risk of bias in studies | 18 | Present assessments of risk of bias for each included study. | Results – "3.2. Study Quality Assessment" and "3.2.1. Risk of Bias Assessment Details" and Figure 2 (domain-level summaries). |
| Results of individual studies | 19 | For all outcomes, present, for each study: (a) summary statistics for each group (where appropriate) and (b) an effect estimate and its precision (e.g. confidence/credible interval), ideally using structured tables or plots. | Results – subsections 3.3 and 3.4, and Tables 1 and 2 (study counts, total n, pooled estimates; not every single |

| | | | |
|---|---|---|---|
| | | | study's individual effect is separately tabulated, but group-level data and some absolute rates are provided). |
| Results of syntheses | 20a | For each synthesis, briefly summarise the characteristics and risk of bias among contributing studies. | Results – "3.3. Technology-Assisted Traditional Venipuncture", "3.4. Population-Specific Approaches", "3.2. Study Quality Assessment", and Table 1 (effect estimates with quality). |
| | 20b | Present results of all statistical syntheses conducted. If meta-analysis was done, present for each the summary estimate and its precision (e.g. confidence/credible interval) and measures of statistical heterogeneity. If comparing groups, describe the direction of the effect. | Results – "3.3.1. Ultrasound-Guided Venipuncture", "3.3.2. Near-Infrared Vein Visualization", "3.4.1. Population-Specific Approaches", "3.5. Economic Impact Analysis" (summary estimates, 95% CIs, heterogeneity via $I^2$ described in methods, direction of effects described throughout). |
| | 20c | Present results of all investigations of possible causes of heterogeneity among study results. | Results – subgroup analyses in "3.3.1. Ultrasound-Guided Venipuncture" (ED vs ward, pediatric vs elderly), "3.3.2. Near-Infrared Vein Visualization" (pediatric, obese, skin |

| | | | |
|---|---|---|---|
| | | | pigmentation, vein depth), and "3.4.1. Population-Specific Approaches" (age groups/sites). |
| | 20d | Present results of all sensitivity analyses conducted to assess the robustness of the synthesized results. | Results – "3.5. Economic Impact Analysis" and "3.5.1. Cost Components Included" (budget-impact and ROI sensitivity) and "3.7. Validation of Proposed Algorithm" (comparison to historical controls); statistical sensitivity analyses for study inclusion/exclusion are mentioned in methods but are summarized narratively. |
| Reporting biases | 21 | Present assessments of risk of bias due to missing results (arising from reporting biases) for each synthesis assessed. | Results – "3.2.1. Risk of Bias Assessment Details" (reporting bias domain: "Reporting bias: Low risk in 85% with comprehensive outcome reporting"), Discussion – "4.4. Limitations of the Present Study" (performance bias, limited long-term outcomes). |
| Certainty of evidence | 22 | Present assessments of certainty (or confidence) in the body of evidence for each outcome assessed. | Results – Table 1 and Table 3 (quality of evidence / evidence level) and narrative in sections 3.3–3.4; |

| | | | |
|---|---|---|---|
| | | | Discussion – section 4 (interpretation of strength of evidence). |
| **DISCUSSION** | | | |
| Discussion | 23a | Provide a general interpretation of the results in the context of other evidence. | Discussion – section 4 (overall interpretation of effectiveness, implementation, economic implications, and clinical impact). |
| | 23b | Discuss any limitations of the evidence included in the review. | Discussion – "4.4. Limitations of the Present Study" (learning curves, operator experience, limited long-term outcomes, inability to blind, residual heterogeneity). |
| | 23c | Discuss any limitations of the review processes used. | Discussion – "4.4. Limitations of the Present Study" (limitations of available data and performance bias; process-specific limitations are discussed but not exhaustively itemized). |
| | 23d | Discuss implications of the results for practice, policy, and future research. | Discussion – sections 4, 4.1–4.3, and Conclusions (clinical implementation, institutional strategy, training priorities, emerging technologies, future research directions). |
| **OTHER INFORMATION** | | | |
| Registration and protocol | 24a | Provide registration information for the review, including register name and registration number, or state that the review was not registered. | Materials and Methods – "2.1. Study Design and Protocol Registration" |

| | | | |
|---|---|---|---|
| | | | (stated that the review followed PRISMA; registration number in PROSPERO or another registry is not explicitly reported, so this is likely "not registered"). |
| | 24b | Indicate where the review protocol can be accessed, or state that a protocol was not prepared. | Materials and Methods – "2.1. Study Design and Protocol Registration" (no separate accessible protocol is cited; effectively "protocol not prepared" or "not publicly available"). |
| | 24c | Describe and explain any amendments to information provided at registration or in the protocol. | Not specifically described; no amendments reported. |
| Support | 25 | Describe sources of financial or non-financial support for the review, and the role of the funders or sponsors in the review. | End of manuscript – "Funding: This research received no external funding." |
| Competing interests | 26 | Declare any competing interests of review authors. | End of manuscript – "Conflicts of Interest: The authors declare no conflicts of interest." |
| Availability of data, code and other materials | 27 | Report which of the following are publicly available and where they can be found: template data collection forms; data extracted from included studies; data used for all analyses; analytic code; any other materials used in the review. | End of manuscript – "Data Availability Statement: Not available." (Template forms, extracted datasets, and code are not publicly deposited.) |
